# Chemically Specific Coherent Raman Imaging of Liquid–Liquid Phase Separation and Its Sequelae

**DOI:** 10.1021/acs.analchem.4c03923

**Published:** 2025-02-07

**Authors:** Alba M. Arbiol Enguita, Laurin Zöller, Teemu Tomberg, Mikko J. Heikkilä, Jukka K. S. Saarinen, Lea Wurr, Tom Konings, Alexandra Correia, Christoph Saal, Jennifer Dressman, Clare J. Strachan

**Affiliations:** † Division of Pharmaceutical Chemistry and Technology, University of Helsinki, Viikinkaari 5E, 00014 Helsinki, Finland; ‡ Fraunhofer Institute of Translational Medicine and Pharmacology, Theodor-Stern-Kai 7, 60596 Frankfurt am Main, Germany; § Department of Chemistry, University of Helsinki, A. I. Virtasen Aukio 1, 00014 Helsinki, Finland; ∥ Boehringer Ingelheim Pharma GmbH & Co., KG, Birkendorfer Strasse 65, 88400 Biberach an der Riss, Germany

## Abstract

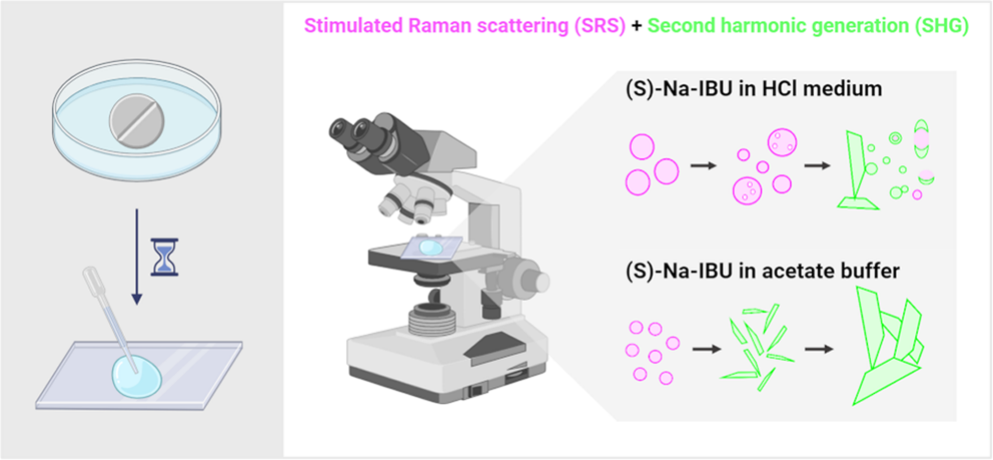

Liquid–liquid phase separation (LLPS) plays a pivotal role in processes ranging from cellular structure reorganization to the formation of crystalline structures in materials science. In the pharmaceutical field, it has been demonstrated to impact drug crystallization and delivery. To date, characterization of LLPS has been limited to nonspatially resolved or nonchemically resolved analyses. In this study, we employed chemically specific stimulated Raman scattering (SRS), combined with second harmonic generation (SHG), for the first time to image crystallization in the presence of LLPS. Using the model compound ibuprofen, we examined the interplay between LLPS and crystallization, and explored the influence of both dissolution medium and enantiomeric form on this behavior. In doing so, we also discovered and partially characterized a new polymorph of (S)-ibuprofen. Our results demonstrate the potential of correlative SRS and SHG for monitoring phase separation and crystallization in real time, giving mechanistic insights into the spatial distribution, chemical composition and structure of phase-separated domains and newly formed crystals. Such insights could benefit not only pharmaceutical development, but also the biomedical, food and chemical sectors.

Liquid–liquid phase separation (LLPS), also called oiling-out, is a ubiquitous phenomenon where a homogeneous supersaturated solution separates into two distinct liquid phases with different compositions. This leads to the formation of a solute-rich and solute-poor phase to reach thermodynamic equilibrium.^1^ LLPS has been reported increasingly during crystallization of both inorganic and biological systems.^2−4^ More recently, LLPS has been observed in the crystallization of small organic molecules in solution, which has implications in the pharmaceutical field, among others.^5−8^ The role of LLPS in crystallization is an area of active research. Conventionally, crystallization has been understood through classical nucleation theory (CNT), which describes the formation of crystals from supersaturated solutions or melts. Recent advances have revealed the existence of alternative, nonclassical crystallization (NCC) pathways, which involve intermediate phases and more complex dynamics.^9^ Several studies suggest that LLPS is an intermediate metastable phase in NCC pathways that acts as a direct precursor of crystallization, and that nucleation occurs within the solute-rich phase.^8,10−15^ Nevertheless, Bonnett et al. pointed out that nucleation can take place in either liquid phase, as both are equally supersaturated.^16^ In this scenario, LLPS would be the result of two components exhibiting (partial) immiscibility, but this phenomenon could occur independently of the nucleation mechanisms. Therefore, the existence of LLPS would not necessarily indicate a nonclassical crystallization pathway.^7,16,17^ Whether crystal nucleation occurs within the solute-rich phase or outside it, there is no doubt that the occurrence of LLPS can significantly influence crystallization processes through several mechanisms. Phase separation might inhibit crystallization by sequestering solute molecules into the solute-rich phase, thereby reducing the effective concentration available for crystal nucleation and growth in the solute-poor phase. On the other hand, increased solute concentration could promote nucleation and/or crystal growth within the solute-rich phase. In addition to crystallization kinetics, LLPS can affect polymorphic outcomes as well as crystal size and shape. These phenomena are of particular importance in pharmaceutical systems as they affect drug release and bioavailability and ultimately the efficacy of therapeutic agents, as well as having a major impact on stability, formulation, processability and manufacturing design. LLPS can be also used as a tool for particle engineering.

The lack of direct experimental evidence of LLPS at the molecular level introduces uncertainty regarding its dynamics and implications for crystallization. Traditionally, atomic force microscopy (AFM) has been used for analysis of classical and nonclassical crystal growth mechanisms.^18,19^ Electron microscopy (EM) techniques have also shed light into NCC mechanisms by revealing the existence of prenucleation clusters with a size of a few tens of nanometers.^14,20−22^ EM reveals valuable nanoscale spatial information not available with other lower-resolution microscopy techniques, but it is not chemically specific. X-ray diffraction methods are generally considered the gold standard in solid-state structural characterization. In particular, small-angle X-ray scattering (SAXS) and wide-angle X-ray scattering (WAXS) have proven to be powerful techniques to investigate nucleation and sensitively detect structural order.^23,24^ However, as nonspatially resolved techniques, they cannot reveal spatial heterogeneity.^17^ LLPS in solution has also been measured using photosensitive turbidity sensors, dynamic light scattering (DLS) or UV–vis spectroscopy, which are all capable of indirectly measuring LLPS based on solution turbidity.^8,25,26^ Computational modeling approaches have potential to give unique insights into molecular processes and predict crystallization kinetic information, which are difficult to obtain empirically and experimentally.^27^ The importance of combining experimental and theoretical studies has been emphasized.^12,28^

In the experimental field, techniques that allow direct visualization and chemical characterization of phase separated and newly formed crystals could give insights into the molecular mechanisms involved in the interplay between these processes. Although the techniques mentioned above have been successfully used to explore LLPS and crystallization, to our knowledge, no technique has been able to simultaneously provide information on the chemical composition and structural dynamics of phase separation and crystallization events.

Vibrational spectroscopy is a powerful tool that can address some of these limitations. Based on molecular vibrations, it is inherently label-free and chemically and structurally specific. The two primary modalities within this field are infrared (IR) spectroscopy and Raman spectroscopy, both of which provide chemical and structural information. These techniques can be combined with microscopy to map the sample composition. However, IR microscopy suffers from some sampling limitations (including strong water absorption) and is often suboptimal for imaging because its diffraction-limited spatial resolution is restricted by the longer wavelengths involved. Spontaneous Raman-based microscopy has been used to explore molecular interactions in different phases.^12,24,29^ Raman can be more suitable than IR for imaging applications due to higher spatial resolution and it is particularly interesting in the pharmaceutical field, since many pharmaceutical compounds are strong Raman scatterers. Confocal Raman microscopy combines the spatial resolution of optical microscopy with the chemical specificity of Raman spectroscopy, allowing for spatially resolved chemical analysis of a heterogeneous sample. However, the spontaneous Raman scattering process is weak, which results in slow image data acquisition particularly when it is used for mapping, precluding analysis of many dynamic heterogeneous systems.

Coherent Raman scattering (CRS) microscopy, including coherent anti-Stokes Raman scattering (CARS) and stimulated Raman scattering (SRS), can overcome these limitations, as it provides the same vibrational information but with higher sensitivity and imaging speed, enabling video-rate imaging.^30−32^ CARS and SRS have been used in the characterization of solid-state materials and dissolution.^33−35^ SRS microscopy has proven to be more sensitive than CARS due to its lack of nonresonant background. Hyperspectral SRS (hsSRS) imaging is particularly interesting as it combines the chemical information on Raman spectroscopy with speed.^36^

Other complementary nonlinear optical (NLO) techniques such as sum frequency generation (SFG) or second harmonic generation (SHG) can be useful to monitor crystallization. SHG is a special case of SFG, in which two photons of the same frequency combine to produce one photon with twice the frequency of the excitation light (2ω). Contrast arises from properties of the crystal structure, as bulk SHG is only possible for noncentrosymmetric structures. This makes it valuable for solid-state analysis of pharmaceuticals due to its ability to provide high-resolution imaging and early detection of crystallization.^37^ Multimodal SRS/SHG imaging enables the simultaneous acquisition of chemical and spatial information, with high sensitivity to structural changes associated with crystalline order (in noncentrosymmetric structures), providing a synergistic approach to characterization. The potential of NLO techniques for *in situ* characterization of crystallization phenomena has been reviewed.^38^

The present study explores the application of SRS imaging and SHG in elucidating LLPS behavior and its sequelae in different dissolution media, using the active pharmaceutical ingredient (API) ibuprofen (IBU) as a model compound. IBU is a widely used nonsteroidal anti-inflammatory API, that is commercially sold in the racemic form (R,S)-IBU, even though (S)-IBU is the therapeutically active form. The API is available as the free acid, as well as salt forms, such as the lysine and sodium salts, with their higher solubility contributing to faster absorption after administration and hence more rapid onset of therapeutic action.

## Experimental Section

### Materials

Racemic IBU ((R,S)-IBU (lot A0311465, purity 99%)) was purchased from Acros Organics B.V.B.A. (New Jersey). S-enantiomeric IBU ((S)-IBU (ReagentPlus, purity 99%)), racemic IBU sodium ((R,S)-Na-IBU (lot BCCG6689, purity ≥98%)) and sodium acetate (C_2_H_3_O_2_Na, batch #119 K0034) were purchased from Sigma-Aldrich (St. Louis). Ethanol (99.5%, Etax Aa) was purchased from Anora Industrial (Finland). Hydrochloric acid (HCl) 1 M and sodium hydroxide (NaOH) 5 M aqueous solutions were purchased from VWR Chemicals BDH (VWR International S.A.S, France).

### Crystallization of S-Enantiomeric IBU-Na

S-enantiomeric IBU-Na ((S)-Na-IBU) was prepared through a straightforward acid–base reaction with NaOH. 500 mg of (S)-IBU was dissolved by adding a few drops of ethanol until complete dissolution was achieved. An equimolar quantity of NaOH (taken from a 5 M aqueous solution of NaOH) was mixed with the (S)-IBU solution. The open vial was placed under a fume hood and crystallization started within 24 h. The salt crystals were dried in vacuum oven for 2 h at room temperature.

### Preparation of IBU Sodium Tablets

Approximately 200 mg of (R,S)-Na-IBU and (S)-Na-IBU were compacted into 13 mm diameter tablets with a hydraulic press (serial number 688, PerkinElmer & Co GmbH, Überlingen, Germany) and a compaction force of approximately 1 kN/cm^2^, without any excipients.

### Dissolution Media

HCl medium (0.1 M) was prepared by adding 63 mL of 1.0 M HCl to 900 mL Milli-Q water and adjusting the pH of the solution to 1.2 (using a Fisherbrand Accumet Basic AB315 Benchtop Laboratory pH/mV Meter, Fisher Scientific Oy, Finland). Acetate buffer with an ionic strength of 0.12 at pH 4.5 was prepared by adding 68 g sodium acetate trihydrate and 40.4 mL concentrated acetic acid to 1 L Milli-Q water. After stirring for 1 h the medium was adjusted to a volume of 5 L and a pH of 4.5.

### Preparation of IBU Amorphous Forms

Amorphous samples of (R,S)-IBU and (S)-IBU were prepared by melting small amounts of the powder on a hot plate at around 80 °C. SRS signals were measured in the forward direction. The amorphous species are referred to throughout the text as (R,S)-IBU AM and (S)-IBU AM. Both correspond to the supercooled melts (above the *T*_g_).^39^

### X-ray Powder Diffraction (XRPD)

The solid-state forms of IBU compounds were analyzed with XRPD using a PANalytical X’Pert Pro MPD diffractometer and Cu–Kα radiation (λ = 1.5419 Å) at 45 kV and 40 mA. Powder samples and dried precipitates were measured in the reflection mode using automatic slits set to 8 mm irradiated/observed length, step size of 0.026°2θ, and angular range of 2.5–44.0°2θ. PIXcel detector was used in 1D mode. Dried precipitates were measured with the parallel beam setup using multilayer mirror and parallel plate collimator in the beam path before the PIXcel detector in 0D mode. Diffractograms were analyzed using HighScore Plus 5.2 (Malvern Panalytical B.V.) software. Nonambient measurements were conducted using the Anton-Paar HTK1200N furnace connected to the same diffractometer, the measurement atmosphere was >99.999% N_2_.

### Spontaneous Raman Spectroscopy

Spontaneous Raman spectra of the materials were measured using a confocal Raman microscope (NT-MDT Ntegra, Russia) equipped with a 532 nm laser (output power ≈20 mW) and a 100× objective (Mitutoyo, Japan). The spectrometer grating had 600 grooves/mm and 600 nm blaze wavelength, giving a spectral resolution of ≈10 cm^–1^ determined as the full width of half-maximum of the Raman peak of silicon at 520.7 cm^–1^. An exposure time of 1 s was used for the acquisition of single spectra. A 50 μm pinhole was used during the spectral acquisition. Samples were placed on top of a #1.5 cover glass and directly measured over the spectral range 225–3680 cm^–1^. OriginPro 2024 (OriginLab Corporation, Massachusetts) was used to preprocess and plot the Raman spectra. Spectra were preprocessed following these steps: adjacent-averaging smoothing (5 points), baseline correction (interpolation method: spline) and min-max intensity normalization.

### Nonlinear Optical Microscopy

Nonlinear optical microscopy was performed using a custom-made coherent Raman microscope, built on a commercial Olympus FV3000 inverted confocal laser scanning microscope by adding a tunable ultrafast dual output laser source (InSight X3+, Spectra-Physics) and (NDD).^200^ It uses a spectral focusing technique (SF-TRU unit, Newport) for stimulated Raman (SRS) and coherent anti-Stokes Raman scattering (CARS) spectroscopy, which renders fast piecewise hyperspectral scans possible over the range 1100–3200 cm^–1^ at a spectral resolution of around 7 cm^–1^.^40^ It is also capable of measuring the SHG or SFG signals either with the NDD PMT detectors, or with the high sensitivity confocal GaAsP PMT detectors with tunable spectral filtering capability. The instrument is operated through both Fluoview FV31S-SW and LabView-based software. For the analyses reported here, the SRS signal was measured by utilizing SRS detection modules from APE-Applied Physics and Electronics in both forward and back scattered directions. The objective used was an Olympus UPLSAPO 60xW1600 NA 1.20 WD 0.28 mm water immersion objective for high spatial resolution. A standard long working distance condenser (IX3-LWUCDA Olympus) with NA 0.55 was used for forward measurements. The lateral (*x*–*y*) spatial resolution was approximately 500 nm.

SRS spectra were recorded by taking image stacks, with one image corresponding to one wavenumber in the spectrum. The SRS spectral range was chosen according to spontaneous Raman data and two different pump wavelengths were required to cover the selected range: 799 nm (2850–3024 cm^–1^) and 793 nm (3027–3099 cm^–1^). The Stokes wavelength was 1045 nm. The laser power at the sample was 27 mW for pump and 78 mW for Stokes. The lateral image size was 512 × 512 pixels, covering a 212 μm × 212 μm area (pixel size was 0.414 μm) with images being collected with a pixel dwell time of 2 μs. With these parameters, the measurement time was around 90 s (84 frames, step size 3 cm^–1^). For each component, suitable areas (also called regions of interest—ROIs) were selected and the average signal over those areas was calculated in order to plot a representative SRS spectrum. For crystalline materials, spectra from five individual crystals were averaged.

SHG was recorded simultaneously in the backscattered direction using a confocal HSD detector of the FV3000 microscope (window 450–550 nm, pinhole open). SHG signal was confirmed by collecting SFG spectra with the pump at 799 nm and Stokes at 1045 nm, by changing the detection range in 10 nm steps from 450 to 550 nm.

Powdered samples of (R,S)-Na-IBU, (S)-Na-IBU, (R,S)-IBU and (S)-IBU were directly measured on top of a #1.5 cover glass and SRS signal was measured in the backscattered direction.

Data preprocessing was performed using an in-house developed MATLAB-based application. SRS spectra were intensity-normalized using min-max normalization and plotted using Origin 2024 (OriginLab Corporation, Massachusetts). Overlay SRS/SHG images were created with Leica Application Suite X software (Leica Microsystems CMS GmbH, Wetzlar, Germany).

### Dissolution Experiments and Nonlinear Optical Imaging

(R,S)-Na-IBU and (S)-Na-IBU tablets were placed on Ø35 mm glass bottom dishes, fitted with a Ø14 mm microwell and a No. 1.5 cover glass (MatTek), and immersed in 3 mL of aqueous buffer.^8^ Measurements of tablet surface and media aliquots were recorded at different time points over approximately 2 h.

**Figure 1:**
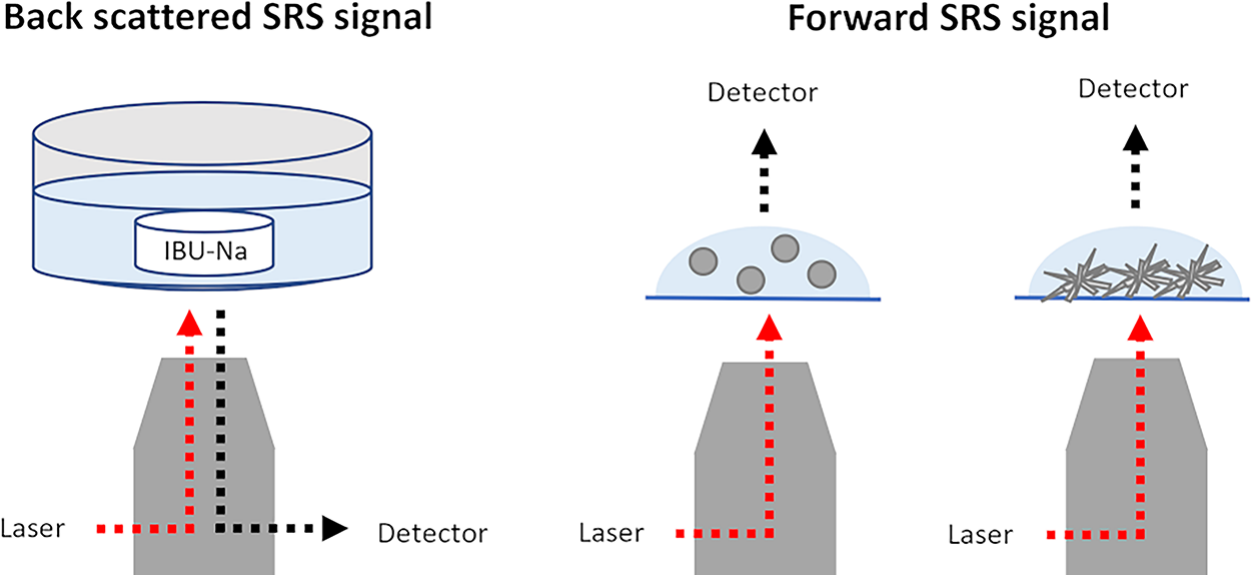
Schematic representation of measurement setup and SRS detection in back- and forward-scattered directions. The IBU sodium tablet is depicted as a white disc, while gray spheres and aggregated needles represent LLPS droplets and crystalline structures, respectively.

For SRS, the scanning setup for the tablet surface and species in liquid media differed. For tablet analyses, the Petri dish containing the tablet and media was positioned in the microscope holder and the signal was measured in the backscattered direction. The tablet surface before buffer addition was also imaged. For examination of species in liquid media and precipitated or solid-like structures, aliquots were collected from the media using a Pasteur pipet or spatula (respectively), deposited on top of a #1.5 cover glass, and analyzed immediately to avoid evaporation of the surrounding liquid. All species in media were measured in the forward direction. Figure 1 provides a schematic representation of the measurement setup and SRS detection scheme. If applicable, SHG signal was also recorded simultaneously as described above.

### Additional Characterization of LLPS and Final Precipitates

LLPS droplets observed during experiments were further characterized using spontaneous Raman spectroscopy. Experiments where solid-like structures had been observed were replicated. The solid materials obtained were collected and dried in a vacuum oven at room temperature to evaporate the media. The resulting products were subsequently characterized using XRPD, spontaneous Raman spectroscopy, and, in some cases, SRS/SHG imaging.

## Results and Discussion

### Characterization of Reference Ibuprofen Forms

XRPD of crystalline IBU samples provided confirmation of the solid-state forms (Figure S1). They were identified as follows (Cambridge Structural Database identifier and space group in brackets): (S)-Na-IBU (KATNOJ, *P*1), (R,S)-Na-IBU (KASVEG, *P*1̅), (S)-IBU (JEKNOC, *P*2_1_) and (R,S)-IBU (IBPRAC, *P*2_1_/c). XRPD from tablet surfaces were recorded by carefully removing sample from the surface with a blade, and those corresponded to the reference salts, proving that there was no conversion due to compaction (data not shown). IBU sodium salts exist as dihydrates in ambient conditions and as the anhydrate upon heating.^41,42^ Both the (R,S)-Na-IBU and (S)-Na-IBU samples started to convert to an anhydrous form immediately after exposure to pure N_2_ atmosphere (Figure S2), confirming their initial dihydrate forms. This is very much in line with the results by Rossi et al.^43^

According to their space groups, (S)-Na-IBU and (S)-IBU are noncentrosymmetric crystal structures, thus SHG active, while the corresponding racemic forms are centrosymmetric crystal structures and therefore non-SHG active. In this study, we used both the racemic and S-enantiomeric salt forms and followed dissolution and potential subsequent crystallization to the free acid, the racemic or enantiomeric form, respectively. The fact that both salt and free acid S-enantiomeric forms are SHG active, whereas amorphous structures do not generate a bulk SHG signal, allowed crystallization to be monitored simultaneously and confirmed using the SHG signal.

**Figure 2:**
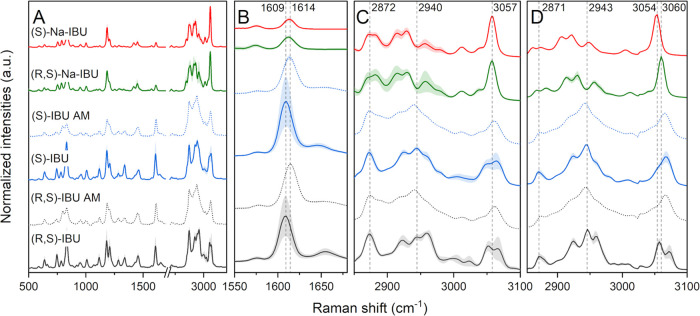
(A–C) Spontaneous Raman spectra of ibuprofen forms in spectral regions (A) 500–3200 cm^–1^, (B) 1550–1680 cm^–1^ and (C) 2850–3100 cm^–1^. (D) SRS spectra of ibuprofen forms in the spectral region 2850–3100 cm^–1^. Lines and shaded areas represent mean ± SD (*n* = 5). Some of the characteristic peaks are marked with gray dashed lines.

We measured spontaneous Raman spectra of the IBU sodium salts, (R,S)-Na-IBU and (S)-Na-IBU, as well as the corresponding crystalline free acids, (R,S)-IBU and (S)-IBU, and their amorphous forms. Figure 2 features spontaneous Raman (Figure 2A–C) and SRS (Figure 2D) spectra of IBU forms. Spectral interpretation was supported by published density functional theory (DFT) calculations for (R,S)-IBU and analysis of Raman spectral variations between crystalline and supercooled liquid phases of the racemic acid.^24,44,45^ The salts, free acids, and their respective amorphous forms can largely be differentiated by peak position and intensity changes, with the most intense peaks in the high wavenumber region (2850–3100 cm^–1^) (Figure 2C).

In the present study, the salts (R,S)-Na-IBU and (S)-Na-IBU exhibited similar spectral profiles with a sharp and intense peak at 3057 cm^–1^ (H–C–C-H antisymmetric stretching), while the free acids (R,S)-IBU and (S)-IBU showed a distinct double peak at around 3057 and 3070 cm^–1^. The salts also exhibited double peaks at around 2872 cm^–1^ (C–H_2_ symmetric stretching) and 2930 cm^–1^ (C–H symmetric stretching). In contrast, the free acids exhibited a sharp single band at around 2872 cm^–1^. The two salts were practically indistinguishable based on their spontaneous Raman spectra. However, the two free acids could be distinguished by the band shapes at 2961 and 3057 cm^–1^; (S)-IBU featured shoulders at these positions, whereas sharp peaks were present for (R,S)-IBU. The spontaneous Raman spectra of amorphous (R,S)-IBU and amorphous (S)-IBU are presented in Figure 2A–C with dotted lines. In general, the amorphous materials exhibited overlapping spectral bands with their corresponding crystalline forms but the peaks were broadened due to the disordered structures and associated nondiscrete variation in molecular conformation and intermolecular interactions. Peak shifts also occurred. For example, the aryl C=C stretching vibration for both (R,S)-IBU and (S)-IBU was blue-shifted at 1614 cm^–1^, when compared to 1609 cm^–1^ (Figure 2B) for the respective crystalline species, in agreement with previous reports.^24,46,47^

SRS spectra in the 2850–3100 cm^–1^ region (Figure 2D) were similar to the corresponding spontaneous Raman spectra. Peak intensities are slightly affected due to spectral focusing during SRS microscopy. The slight peak shifts of approximately 3 cm^–1^ between the two salts around 3057 cm^–1^ are due to spectral focusing calibration, which is not entirely accurate. The amorphous forms exhibited broadened SRS modes compared to the sharper band characteristic of crystalline materials.

The influence of crystal orientation on the SRS and SHG signals was investigated. SRS spectra presented in Figure 2D are the means (solid line) ± SD (shaded areas) of spectra from different individual crystals with different orientations. While crystal orientation does not influence peak position, it does affect peak intensity. This is important as it gives a measure of confidence when looking at the spectral shapes and assigning forms. For (S)-Na-IBU and (S)-IBU, the SHG signal overlaps with SRS, but some crystal orientations show higher SHG (verified by rotating the laser polarization 90 deg, data not shown). Orientation effects of crystals in nonlinear optical microscopy have been described elsewhere.^48,49^

### NLO Imaging of IBU Forms

**Figure 3:**
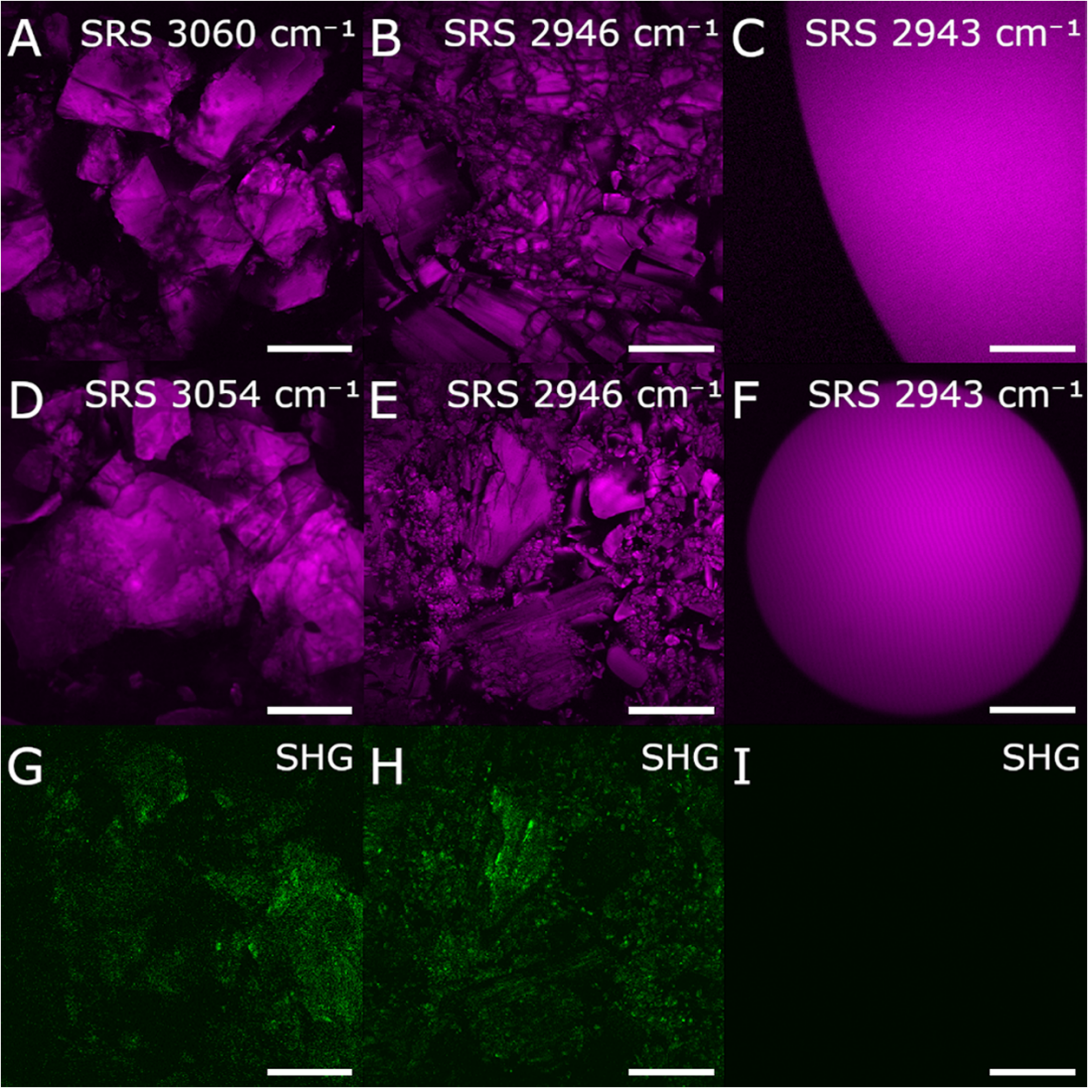
(A–F) SRS images of (A) (R,S)-Na-IBU, (B) (R,S)-IBU, (C) (R,S)-IBU AM, (D) (S)-Na-IBU, (E) (S)-IBU and (F) (S)-IBU AM, at the Raman shift corresponding to the highest signal in each spectrum. (G–I) Corresponding SHG images of (G) (S)-Na-IBU, (H) (S)-IBU and (I) (S)-IBU AM. Scalebars: 50 μm.

The high wavenumber region, 2850–3100 cm^–1^, was selected for SRS imaging. SRS images generated using the most intense peak for each of the forms are shown in Figure 3A–F. SHG signals (Figure 3G–I) were recorded for (S)-Na-IBU, (S)-IBU and amorphous (S)-IBU. (R,S)-Na-IBU, (S)-Na-IBU and the corresponding free acids, (R,S)-IBU and (S)-IBU, are all roughly prismatic but largely polycrystalline. (R,S)-IBU AM and (S)-IBU AM formed spherical droplets with intense SRS signal. The amorphous state of the (S)-IBU AM was further confirmed with the absence of any SHG signal.

The SFG spectrum contained the predicted SFG peak at 453 nm and the SHG of the Stokes was at 522 nm (data not shown). The positions of the peaks and the absence of a broad background signal confirmed that the detected signal originated from SFG/SHG and not from two-photon fluorescence.

### Chemically Specific Imaging of LLPS and Crystallization

Several studies have used indirect detection and nonchemically specific microscopy to suggest that supersaturated solutions of IBU can undergo LLPS through spinodal decomposition.^15,22,50^ In the current study, the IBU sodium salts, (R,S)-Na-IBU and (S)-Na-IBU, were used to generate supersaturated solutions with respect to the corresponding amorphous free-acid forms of IBU and thus create conditions allowing LLPS formation and crystallization.

**Figure 4:**
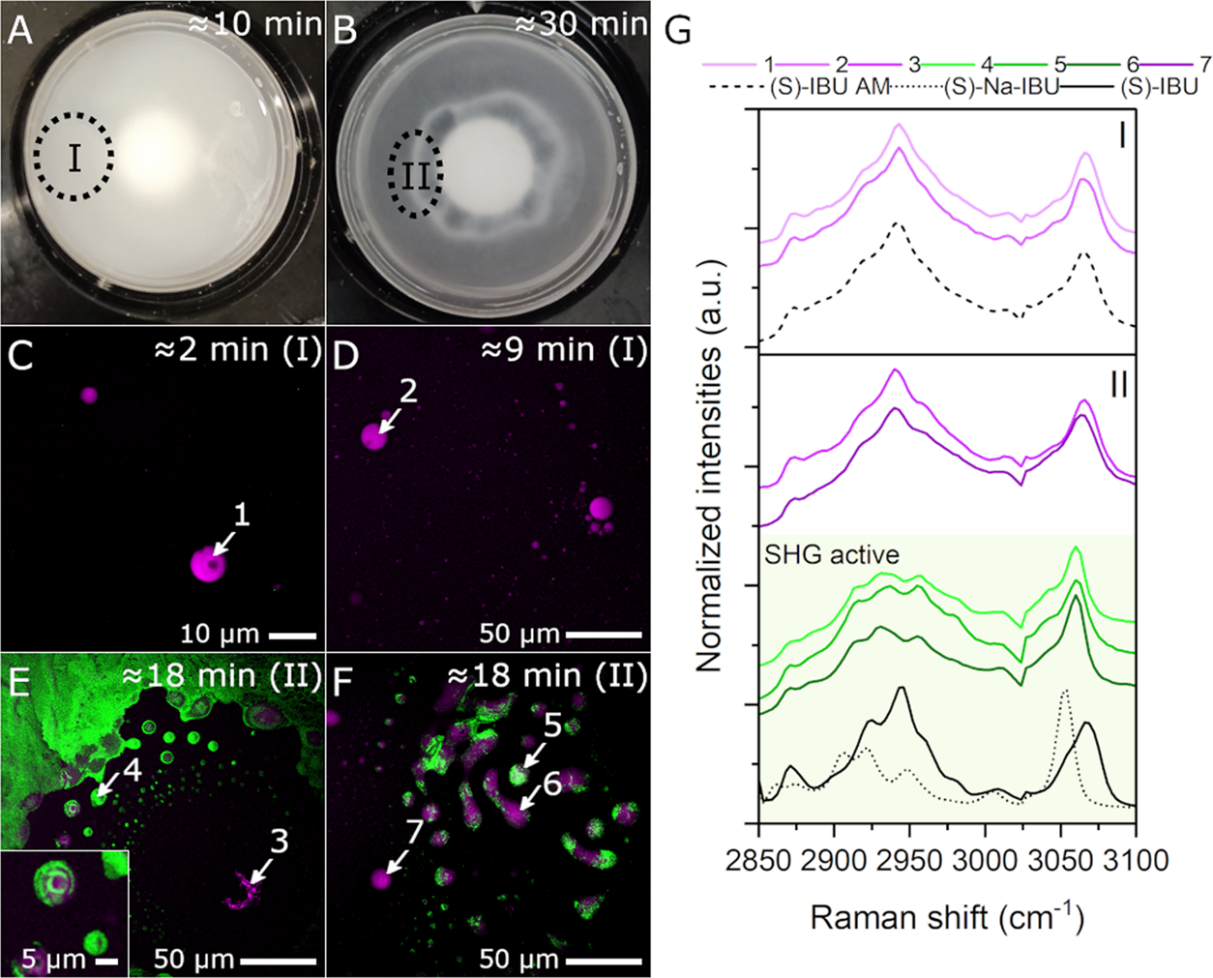
Phase behavior following (S)-Na-IBU addition to HCl medium (Scenario 1). (A, B) Pictures taken with a camera at time points ≈10 min and ≈30 min, respectively. Zones representing different phase behavior are marked with dotted ellipses as zones I and II. (C–F) SRS/SHG overlay images from aliquots collected during crystallization (SRS image contrast is provided using the SRS intensities at the Raman shifts with the strongest signal for each phase). The inset in (E) is a close-up of droplets exhibiting crystallinity. The approximate time point and zone of collection are indicated in each overlay image. White arrows with numbers represent regions where SRS spectra were extracted. (G) SRS spectra of areas indicated with white arrows in (C–F) (colored lines) and reference spectra of (S)-IBU AM (black dashed line), (S)-Na-IBU (black dotted line) and (S)-IBU (black solid line) for comparison. Spectra are divided into two boxes according to the zone where aliquots were collected. Spectra from SHG active species are within a light green box.

**Figure 5:**
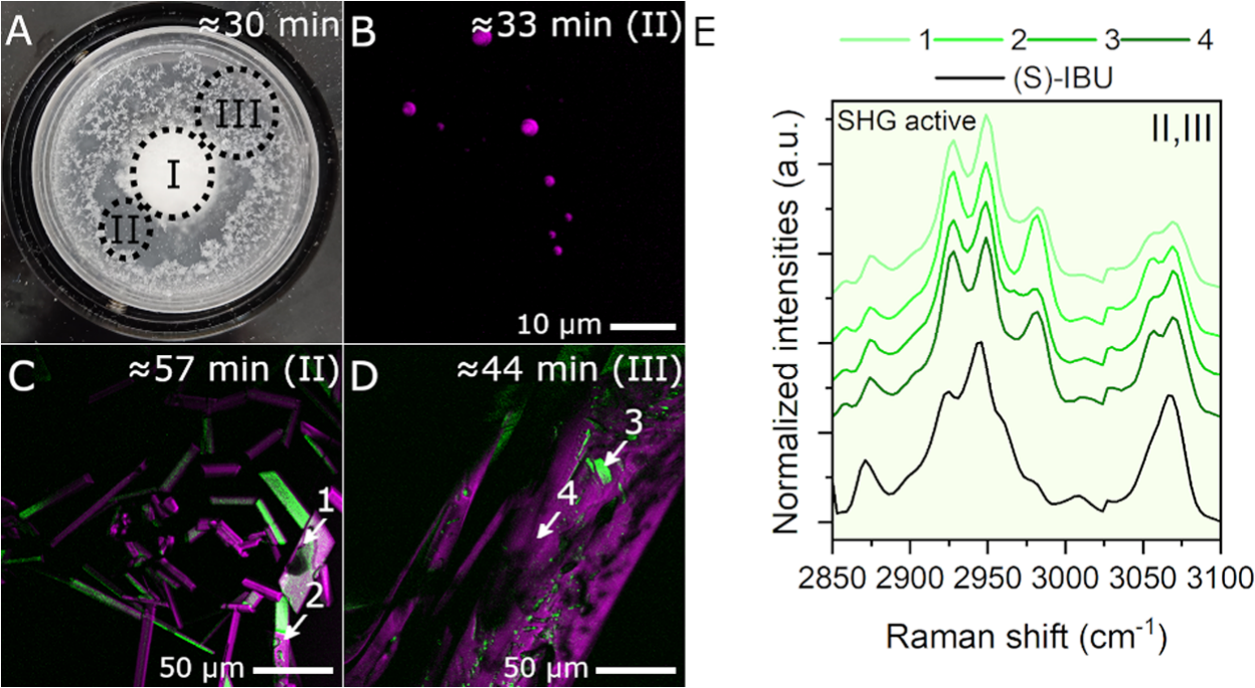
Phase behavior following (S)-Na-IBU addition to acetate buffer (Scenario 2). (A) Picture taken with a camera at time point ≈30 min, respectively. Zones representing different phase behavior are marked with dotted ellipses as zones I, II and III. (B–D) SRS/SHG overlay images from aliquots collected during crystallization (SRS image contrast is provided using the SRS intensities at the Raman shifts with the strongest signal for each phase). The approximate time point and zone of collection are indicated in each overlay image. White arrows with numbers represent regions where SRS spectra were extracted. (E) SRS spectra of areas indicated with white arrows in (B–D) (colored lines) and reference spectra of (S)-IBU (black solid line) for comparison. Spectra from SHG active species are within a light green box.

Four scenarios were investigated, representing the enantiomeric and racemic forms of the sodium salts and the two media: (1) (S)-Na-IBU in HCl medium; (2) (S)-Na-IBU in acetate buffer; (3) (R,S)-Na-IBU in HCl medium; and (4) (R,S)-Na-IBU in acetate buffer. At least two replicates per scenario were performed, with results from representative studies presented. The corresponding Figures ((1) Figure 4; (2) Figure 5; (3) Figure S3 and (4) Figure S4) include one or two pictures taken with a camera during the experiments (except Scenario 4, in which observations corresponded to those in Scenario 2). Dashed ellipses in these pictures are used to mark zones (I, II and III), each with different appearance. During the experiment (which starts with dissolution medium addition), aliquots from these zones were withdrawn, placed on a coverslip, and measured. Times indicated in the images correspond to the time points where the aliquots were collected. The observed phenomena occurred during overlapping temporal ranges. As such, the provided time points serve only as an approximate indication of the observed progression. SRS images alone (in the case of the non-SHG active forms) or SRS/SHG overlay images (for SHG active forms) are shown (magenta is SRS signal at the most intense peak, green is SHG), alongside extracted spectra from selected regions. Tablet surfaces were systematically monitored as well (at time point 0 min and along the experiment). There was no crystallization on the tablet surface (data not shown).

### (S)-Na-IBU in HCl Medium (Initial pH 1.2)

#### Scenario 1

Dissolution of (S)-Na-IBU in HCl medium (at initial pH 1.2) and subsequent crystallization was followed using SRS microscopy combined with SHG (Figure 4). Almost immediately after adding the medium, the liquid surrounding the tablet exhibited high turbidity (Figure 4A, zone I), suggestive of LLPS formation.^8,12,22^ This phase separation (spinodal decomposition) was due to formation of dissolved un-ionized free acid in the acidic medium, upon which the solution became supersaturated with respect to the amorphous form of un-ionized free acid, (S)-IBU. After approximately 30 min, an opaque white phase formed a halo at a distance from the tablet surface (Figure 4B, zone II). The halo formation is likely a consequence of a concentration and pH gradient from the tablet surface. The LLPS droplets and subsequent opaque white phase were subjected to analysis using SRS/SHG microscopy.

Aliquots from turbid media at 2 min (Figure 4C) showed spherical droplets about 5–10 μm in diameter. The size of the droplets increased with time. We observed that this was due, at least partly, to coalescence, which is consistent with the observations of Bonnett et al.^16^ After 9 min, the droplet size was about 10–20 μm (Figure 4D). SRS spectra of the observed species are found in Figure 4G. SRS spectra of small droplets at time points 2 and 9 min (spectra 1 and 2) suggest that they were enriched with amorphous IBU. This is supported by the fact that no SHG activity was observed. These observations are in agreement with those reported by Mani et al.^24^ Halo aliquots, collected from zone II at time point ≈18 min, consisted of spherical precipitates with very strong SHG activity, indicating noncentrosymmetric crystallinity (Figure 4E,F). The crystallinity was detectable within the separated phase but appeared initially more intense at the edges of the droplets, which suggests that the interfaces between phase-separated domains could serve as preferential sites for initial crystallization. Nevertheless, the SHG signal was also intense within the interior of some of the droplets.

SRS spectra from several SHG active structures were extracted (spectra 4–6) and found to be similar to one another. However, they resembled neither the (S)-IBU free acid nor the salt, which suggests the formation of a new polymorph, crystallizing within the LLPS droplets. To our knowledge, no other polymorphs of (S)-IBU have been reported to date. A polymorph of (R,S)-IBU, which can be obtained by recrystallization of liquid racemic IBU was reported by Dudognon et al.,^51^ but it has a melting point under room temperature (between 288 and 293 K). Further solid-state characterization of the observed form was therefore performed with spontaneous Raman and XRPD (the results of which are reported the section Additional Characterization of LLPS and Final Precipitates, *vide infra*). Over time, the visible halo redissolved. After about 2 h, the tablet completely dissolved, and no discernible solid or separated phase was evident in the medium. This was due to the pH increasing from 1.2 to about 7 (measured using pH indicator paper) and thus the IBU was again ionized, and the solubility was no longer exceeded.

### (S)-Na-IBU in Acetate Buffer (Initial pH 4.5)

#### Scenario 2

The phase separation and crystallization behavior of (S)-Na-IBU in acetate buffer, at the higher initial pH of 4.5, was also monitored (Figure 5). Based on visual observation, in this case, the medium did not become as turbid as in Scenario 1. Turbidity increased more slowly, probably due to the higher solubility at the higher pH (Figure 5A, zone II). SRS images showed phase separated droplets with a size of about 2 μm (Figure 5B). SRS spectra extracted from these droplets resembled that of the amorphous (S)-IBU (data not shown) and the droplets were not SHG active. It was also possible to image small (1–2 μm wide and 10–20 μm long) rectangular and needle-shaped SHG active crystals within the medium at later time points (Figure 5C). After about 30 min, a precipitate floating on the medium started to appear (zone III). The precipitate consisted of larger SHG active crystals (about 50 μm wide and more than 200 μm long) (Figure 5D). SRS spectra of both the small (spectra 2 and 3) and large (spectra 4 and 5) crystals resembled that of (S)-IBU (Figure 5E). Notably, some of the observed crystals showed high SHG signal while others did not, due to different crystal orientations. All newly formed crystals showed consistent SRS spectra, regardless of their SHG activity. The crystals grew at the expense of LLPS. Once the first free acid crystals appeared, all LLPS in the medium quickly disappeared (see video in Supporting Information). Interestingly, droplets showed fast movement around the tablet. These convection events are likely driven by Marangoni effects, which arise from surface tension gradients caused by phase differences within the suspension.^52^

### (R,S)-Na-IBU in HCl Medium (Initial pH 1.2)

#### Scenario 3

(R,S)-Na-IBU in HCl medium (Figure S3) showed visually similar behavior to (S)-Na-IBU (described in Scenario 1, Figure 4), with initial turbidity (Figure S3A) and subsequent formation of a halo around the tablet (Figure S3B). Over time, this halo vanished, and the tablet completely dissolved (due to the pH increasing, as described in Scenario 1). LLPS droplets in the medium were spherical and their size increased over time (from around 5–10 μm at 3 min, up to 20–40 μm after 21 min) (Figure S3C–D). SRS images from halo aliquots showed enlarged spherical and elliptical aggregates of sizes up to 100 μm (Figure S3E,F) but, contrary to Scenario 1, crystalline structures were not detected. Upon analysis of the measured spectra (Figure S3G), all LLPS droplets contained amorphous (R,S)-IBU.

The mechanism of LLPS is spinodal decomposition, which leads to the formation of a drug-rich phase and a drug-poor phase. As the system progresses toward thermodynamic equilibrium, the concentration difference between the two phases becomes more pronounced. When the phase-separated droplets are sufficiently large, some of the solvent is ejected internally within the drug-rich droplets (instead of from the surfaces). The round holes (without SRS signal) that appear in the big LLPS domains as revealed by SRS are presumably pockets of solvent resulting from solvent ejection.

### (R,S)-Na-IBU in Acetate Buffer (Initial pH 4.5)

#### Scenario 4

The immersion of the (R,S)-Na-IBU tablet in acetate buffer showed the same behavioral pattern as the corresponding enantiomer (Scenario 2, Figure S4). LLPS (Figure S4A) was followed by the formation of a crystalline phase within the drug-poor phase (Figure S4B–C). Based on the SRS spectra (Figure S4D), the defined drug-rich droplets contained the amorphous form of (R,S)-IBU (data not shown) and all crystals were identified as crystalline (R,S)-IBU (spectra 1–5). Similarly, new crystals grew at the expense of LLPS droplets.

### Overview of Phase Behavior in Different Conditions

**Figure 6:**
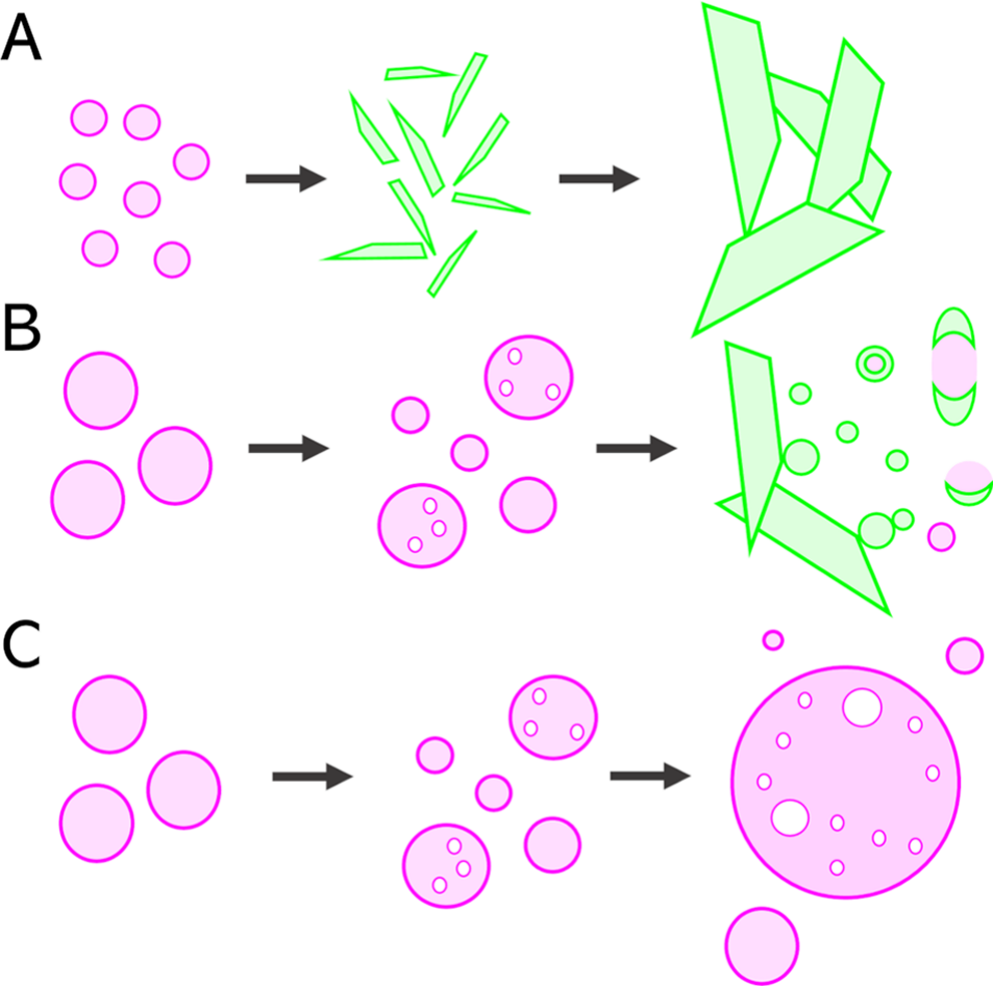
Schematic representation of phase behavior in Scenarios 1–4. Light pink spherical structures represent LLPS droplets (consisting of amorphous IBU), whereas crystalline structures are depicted in light green. White holes within larger LLPS droplets represent solvent pockets. (A) (S)-Na-IBU and (R,S)-Na-IBU in acetate buffer (Scenarios 2 and 4, respectively). Small LLPS droplets appeared during the first minutes. Subsequently, small and then big crystals of (S)-IBU or (R,S)-IBU grew at the expense of the LLPS droplets. (B) (S)-Na-IBU in HCl medium (Scenario 1). Initial LLPS were bigger and more stable compared to those observed in Scenarios 2 and 4. The right-most illustration depicts observations within the halo, where spherical crystalline precipitates were visible. Interestingly, some fused together and also with other crystalline structures. (C) (R,S)-Na-IBU in HCl medium (Scenario 3). The initial behavior was similar to Scenario 1, but in this case the halo consisted of large LLPS droplets.

Our coherent Raman imaging, combined with SHG, revealed LLPS and *in situ* crystallization from supersaturated solutions, after dissolution of (R,S)-Na-IBU and (S)-Na-IBU in HCl medium and acetate buffer, at starting pHs of 1.2 and 4.5, respectively. Phase behavior was different in the two media, which can be attributed to different degrees of ionization, solubility and supersaturation. A schematic representation of the phase behavior under different conditions is shown in Figure 6. Both the S-enantiomeric and racemic salts behaved similarly in acetate buffer (Scenarios 2 and 4, respectively). In both cases, we observed the formation of liquid droplets enriched with amorphous IBU, representing LLPS, followed by the formation of at first small, and then large, needle-shaped crystals (Figure 6A). SRS spectra of the newly formed crystals resembled those of the respective free acids. In the case of S-enantiomeric salt (Scenario 2), SHG activity confirmed the crystallinity of the final product. Figure 6B–C depict behavior of (S)-Na-IBU and (R,S)-Na-IBU in HCl medium (Scenarios 1 and 3, respectively). The common denominator in both scenarios was the initial formation of LLPS droplets that gradually got bigger, and the subsequent formation of a white opaque phase forming a halo around the tablet. Interestingly, for (S)-Na-IBU, this halo contained a crystalline final product with intense SHG activity (Figure 6B). Based on SRS spectra, we speculated this could be a new polymorph of (S)-IBU crystallized under these specific conditions. No crystal structures were identified for (R,S)-Na-IBU, and the halo instead consisted mostly of large LLPS droplets (Figure 6C). It is worth noting that, in acetate buffer, crystallization (for both salts) happened in the drug-poor phase. This is contrary to the observations in HCl medium (for (S)-Na-IBU), where crystal growth was visualized within the drug-rich phase. The potential for crystallization within the drug-poor phase also aligns with the findings of Bonnett et al.^16^ No dissolved drug signal was detected in the drug-poor phase for any dissolution medium (below limit of detection).

The possibility of laser-induced nucleation was considered.^53,54^ We do not believe that the observations were affected by this phenomenon because imaging was conducted across different areas, rather than repeatedly focusing on the same area for extended periods of time.

The findings presented in this study highlight the unique ability of our combined SRS and SHG approach to provide chemically specific, spatially resolved, and real-time insights into chemical composition and structural dynamics of phase separation and crystallization.

### Additional Characterization of LLPS and Final Precipitates

According to SRS/SHG microscopy, the LLPS droplets were always enriched with amorphous IBU. More detailed peak position analysis with spontaneous Raman spectroscopy was performed for additional confirmation. As discussed earlier, detection of the aryl C=C stretching vibration around 1609 and 1614 cm^–1^ could help to differentiate between amorphous and crystalline species. Spontaneous Raman was used to measure the full spectra of LLPS in the HCl medium. The acquisition of spectra from LLPS formed in acetate buffer was not possible due to the smaller size and rapid redissolution of the LLPS, as described in Scenarios 2 and 4. Spontaneous Raman spectra of LLPS in the HCl medium (Figure S5) matched those of the respective amorphous references from melted (R,S)-IBU and (S)-IBU, with broadened peaks. In the fingerprint region, a peak at 1614 cm^–1^ was blue-shifted compared to that of the crystalline form at 1609 cm^–1^. Solid-like structures underwent additional solid-state analysis. Figure S6 shows spontaneous Raman and XRPD data from the precipitate obtained in Scenario 4 ((R,S)-Na-IBU in acetate buffer). The spectra and XRPD pattern match those of the reference form (R,S)-IBU. For the (S)-Na-IBU salt, it was not possible to measure exactly the same final products as those obtained from the experiments shown in SRS/SHG imaging. Therefore, replicate experiments of Scenarios 1 and 2 were performed for more detailed solid-state analysis of the final products. The replicate from Scenario 1 ((S)-Na-IBU in HCl medium) showed the same halo formation. The aliquot collected from the halo exhibited crystallinity, with a SRS spectrum distinct from that of the (S)-IBU reference. The new crystals featured a peak at 2928 cm^–1^, red-shifted compared to that of the (S)-IBU reference form (hereafter referred to as (S)-IBU (form I)), as well as an additional shoulder at 2874 cm^–1^ (Figure S7C). We believe these spectra represent a new polymorph of (S)-IBU, which we denote as (S)-IBU (form II). This form was also detected in the precipitate obtained from a replicate of Scenario 2 ((S)-Na-IBU in HCl medium). SRS microscopy, combined with classical least-squares (CLS) analysis of the image spectral data, demonstrated that the precipitate contained a mixture of forms I and II, while the halo in the HCl medium was pure form II (Figure S7A,B). Form II was SHG active (data not shown), which indicates that the crystals are noncentrosymmetric.

Spontaneous Raman spectra and XRPD patterns from the same samples were recorded for further solid-state confirmation. Figure S8A shows spontaneous Raman spectra from the halo in the HCl medium and the precipitate in acetate buffer, along with reference IBU forms, for comparison. The spectra revealed identical peak shifts for form II in the high wavenumber region as described for SRS microscopy. Additionally, the peak at 1609 cm^–1^ for form I shifted to 1614 cm^–1^ for form II. XRPD analysis confirmed that the halo in the HCl medium consisted of pure (S)-IBU form II. Spontaneous Raman spectroscopy (Figure S8A) and XRPD (Figure S8B) confirmed that the precipitate in acetate buffer was a mixture of (S)-IBU forms I and form II.

The experiments described in Scenarios 1 and 2 and their respective replicates were compared with regard to the spectra of their final products (Figure S9). The spherical droplets obtained in Scenario 1 displayed spectra that did not resemble any known (S)-IBU forms, which may indicate yet another new polymorph. Nevertheless, we did not manage to confirm this with additional solid-state analysis. The precipitate in Scenario 2 appeared to contain only (S)-IBU form I, yet the replicate showed a mixture of forms I and II, as discussed above. This suggests that the crystallization might have occurred differently in the replicate compared to the experiment described in SRS/SHG microscopy. Future work could include synchrotron-based *in situ* X-ray diffraction for more detailed *in situ* solid-state analysis.

## Conclusions

In this study, hyperspectral SRS microscopy combined with SHG was used to gain a deeper understanding of the spatial distribution, chemical and solid-state composition, and temporal evolution of phase-separated domains of IBU in supersaturated aqueous systems. To our knowledge, this is the first study involving concomitant, *in situ*, chemically and structurally specific imaging of LLPS and various other structures at sequential phase separation steps. The results show the potential advantages of *in situ* SRS imaging combined with SHG over conventional techniques for real time monitoring of LLPS and crystallization processes in dissolution environments, with the potential to provide deeper insights into complex phase separation and crystallization events in the pharmaceutical and other sectors, such as the chemical and food industries.

## Supporting Information Available

Video recorded during experiment reported in Scenario 2 (Figure 5) (MP4)
XRPD of IBU forms and reference patterns from CSD database, nonambient XRPD measurements, figures corresponding to phase behavior of racemic IBU sodium salts in HCl medium and acetate buffer, additional characterization of LLPS and final precipitates (with SRS, spontaneous Raman spectra and XRPD) (PDF)
