# Supplementary material for: Chemically Specific Coherent Raman Imaging of Liquid–Liquid Phase Separation and Its Sequelae
Source: Anal Chem. 2025 Feb 7;97(6):3242–52. doi: 10.1021/acs.analchem.4c03923 (PMC11840799; doi:10.1021/acs.analchem.4c03923)
Supplement: Supplementary file 2 — ac4c03923_si_002.pdf [file ac4c03923_si_002.pdf]

SUPPORTING INFORMATION

Chemically-specific coherent Raman imaging of liquid-liquid phase separation and its sequelae

Alba M. Arbiol Enguita[1‡*], Laurin Zöller[2‡], Teemu Tomberg[1], Mikko J. Heikkilä[3], Jukka K.S. Saarinen[1], Lea Wurr[1], Tom Konings[1], Alexandra Correia[1], Christoph Saal[4], Jennifer Dressman[2], Clare J. Strachan[1]

[1]Division of Pharmaceutical Chemistry and Technology, Viikinkaari 5E, 00014 University of Helsinki, Finland

[2]Fraunhofer Institute of Translational Medicine and Pharmacology, Theodor-Stern-Kai 7, 60596 Frankfurt am Main, Germany

[3]Department of Chemistry, A. I. Virtasen aukio 1, 00014 University of Helsinki, Finland

[4]Boehringer Ingelheim Pharma GmbH & Co. KG, Birkendorfer Strasse 65, 88400 Biberach an der Riss, Germany

[‡]These authors contributed equally to this work

[*]Corresponding author (alba.arbiol@helsinki.fi)

Table of contents

X-ray powder diffraction (XRPD) of ibuprofen forms **S-2**

Figure S1. XRPD of ibuprofen forms and reference patterns from CSD database S-2

Figure S2. Non-ambient XRPD measurements of (S)-Na-IBU and (R,S)-Na-IBU S-3

Additional experiments with racemic ibuprofen sodium salts **S-4**

Figure S3. Phase behavior following (R,S)-Na-IBU addition to HCl medium (*Scenario 3*) S-4

Figure S4. Phase behavior following (R,S)-Na-IBU addition to acetate buffer (*Scenario 4*) S-5

Additional characterization of LLPS and final precipitates **S-6**

Figure S5. Spontaneous Raman spectra of LLPS in crystallization experiments of (S)-Na-IBU and (R,S)-Na-IBU in HCl medium (*Scenarios 1* and *3*) S-6

Figure S6. Spontaneous Raman spectra and XRPD of collected precipitate in crystallization experiments of (R,S)-Na-IBU in acetate buffer (*Scenario 4)* S-7

Additional characterization of S-IBU form II **S-8**

Figure S7. SRS images (based on classical least squares (CLS) analysis of image spectra), corresponding to the halo in HCl medium and precipitate in acetate buffer S-8

Figure S8. Spontaneous Raman spectra and XRPD diffractograms of collected samples corresponding to the halo in HCl medium and precipitate in acetate buffer S-9

Figure S9. SRS spectrum of a crystalline droplet from the halo in HCl medium (*Scenario 1*), as well as spectra of reference (S)-IBU forms I and II S-10

X-ray powder diffraction (XRPD) of ibuprofen forms

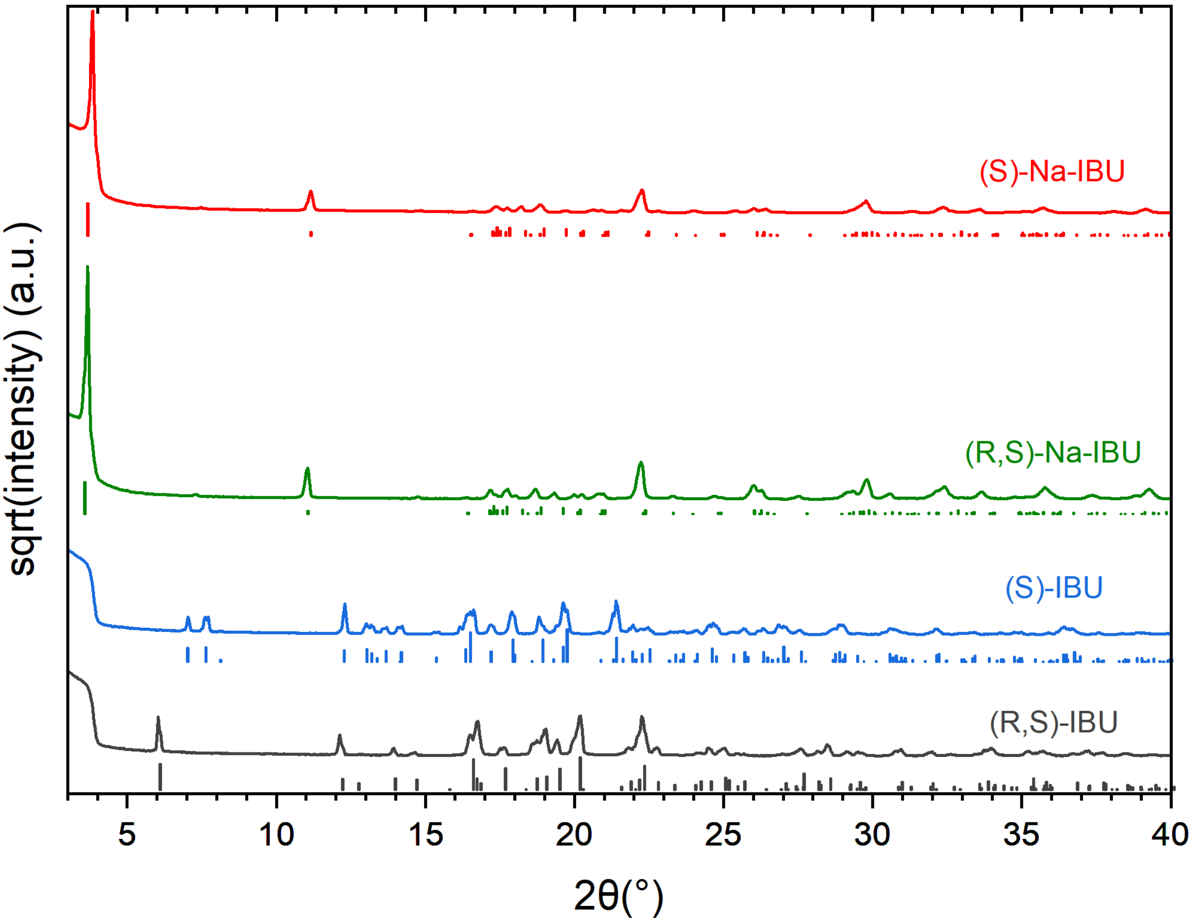

Figure S1. Continuous lines represent the measured X-ray diffractograms of (S)-Na-IBU, (R,S)-Na-IBU, (S)-IBU and (R,S)-IBU. The column graphs below each diffractogram represent the reference positions and intensity ratios of the reflections, and from top to bottom they refer to the CSD identifiers (space group given in brackets): KATNOJ (P 1), KASVEG (P -1), JEKNOC, (P 2_1), and IBPRAC (P 2_1/c). The intensity axis is shown as the square root to make low intensity reflections more visible. High intensity at the beginning of each measurement is due to air scattering.

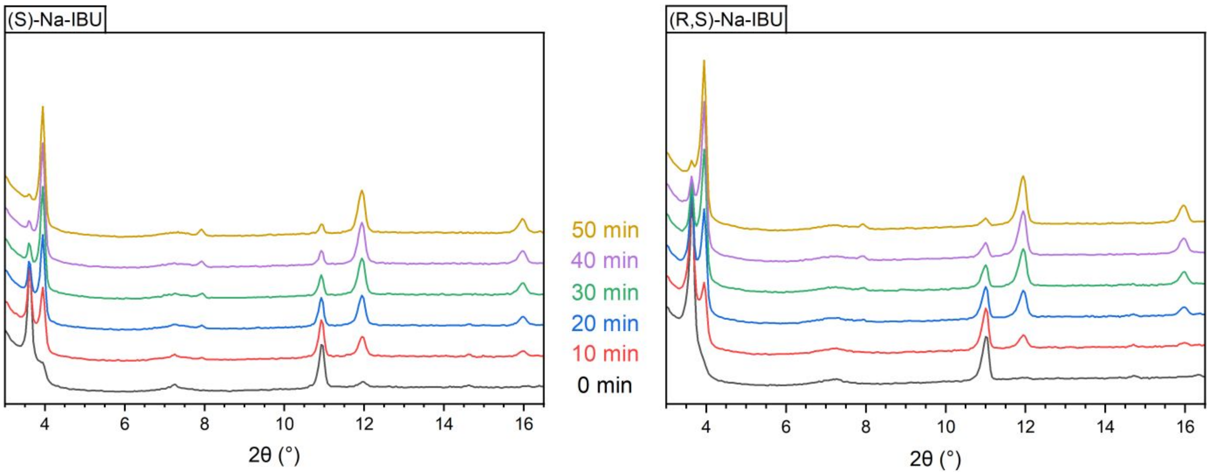

Figure S2. XRPD diffractograms of (S)-Na-IBU and (R,S)-Na-IBU samples measured inside the Anton Paar HTK1200N furnace. The temperature was constant at 25 °C and the atmosphere inside was >99.999 % N_2. The 0 min time point is the beginning of the first measurement immediately after inserting the sample inside the chamber and N_2 atmosphere.

Additional experiments with racemic ibuprofen sodium salts

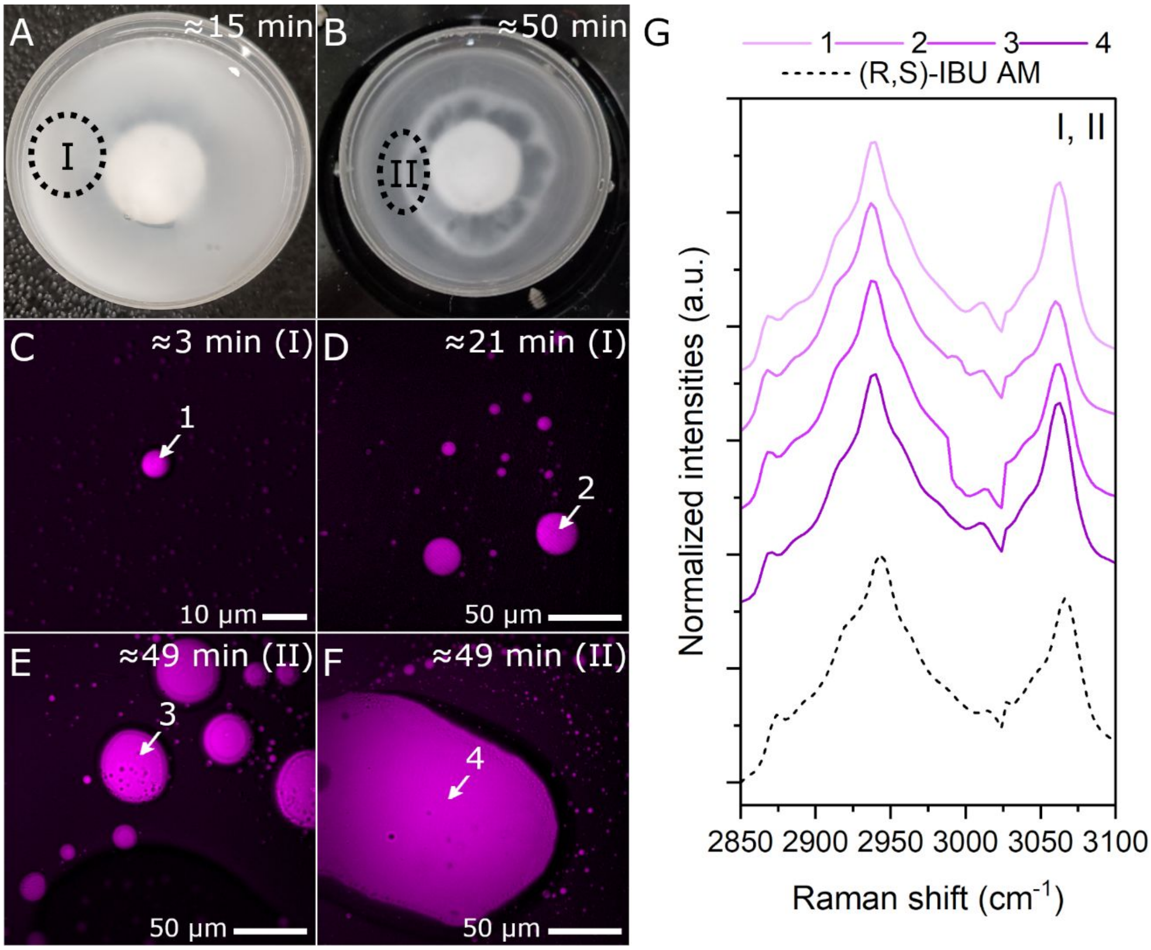

Figure S3. Phase behavior following (R,S)-Na-IBU addition to HCl medium (*Scenario 3)*. (A-B) Pictures taken with a camera at time points ≈15 min and ≈50 min, respectively. Zones representing different phase behaviour are marked with dotted ellipses as zones I and II. (C-F) SRS images from aliquots collected during crystallization (SRS image contrast is provided using the SRS intensities at the Raman shifts with the strongest signal for each phase). The approximate time point and zone of collection are indicated in each image. White arrows with numbers represent regions where SRS spectra were extracted. (G) SRS spectra of areas indicated with white arrows in (C-F) (color lines) and reference spectra of (R,S)-IBU AM (black dashed line) for comparison.

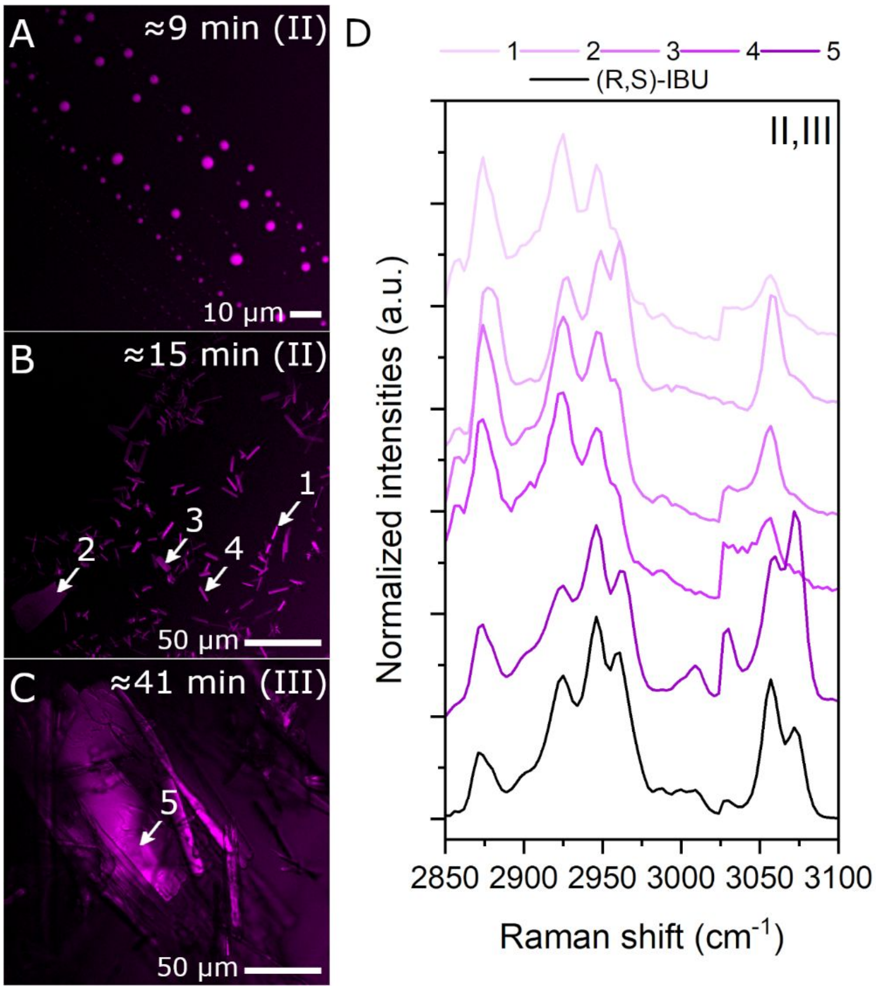

Figure S4. Phase behavior following (R,S)-Na-IBU addition to acetate buffer (*Scenario 4)*. The progression visually looked the same as shown in *Scenario 2* (Fig. 5A). (A-C) SRS images from aliquots collected during crystallization (SRS image contrast is provided using the SRS intensities at the Raman shifts with the strongest signal for each phase). The approximate time point and zone of collection are indicated in each image. White arrows with numbers represent regions where SRS spectra were extracted. (D) SRS spectra of areas indicated with white arrows in (A-C) (color lines) and reference spectra of (R,S)-IBU (black solid line) for comparison.

Additional characterization of LLPS and final precipitates

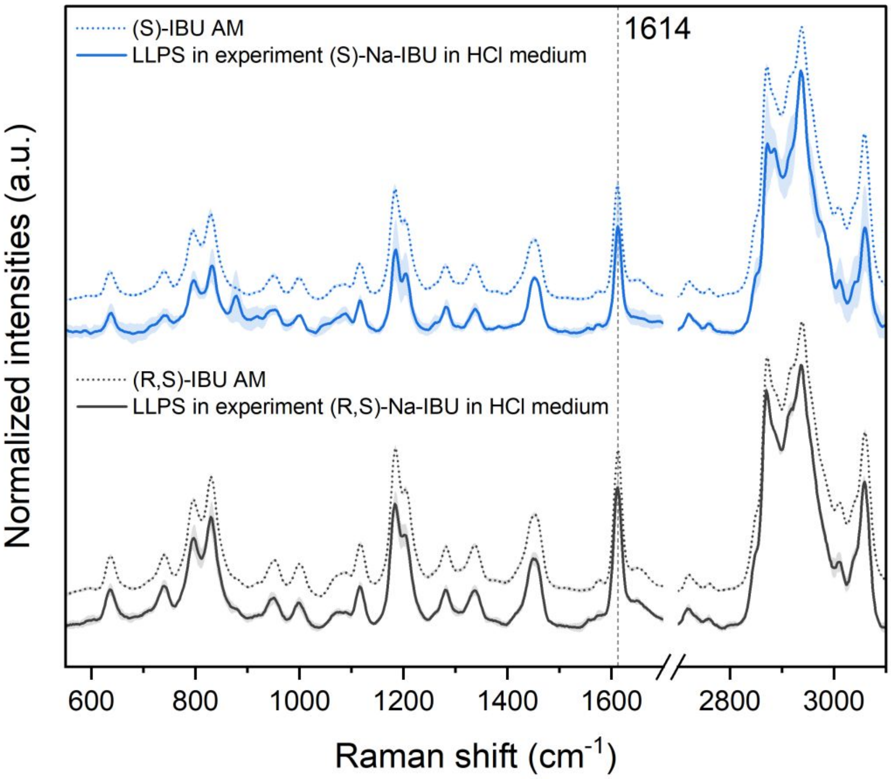

Figure S5. Spontaneous Raman spectra (mean ± SD, n=5) of LLPS in crystallization experiments of (S)-Na-IBU (blue solid line) and (R,S)-Na-IBU (black solid line) in HCl medium, corresponding to *Scenarios 1* and *3*, respectively. Spectra of (S)-IBU AM and (R,S)-IBU AM are presented as dotted lines for reference. The dashed vertical line indicates the peak at 1614 cm^{-1}, characteristic for the amorphous forms of (S)-IBU and (R,S)-IBU.

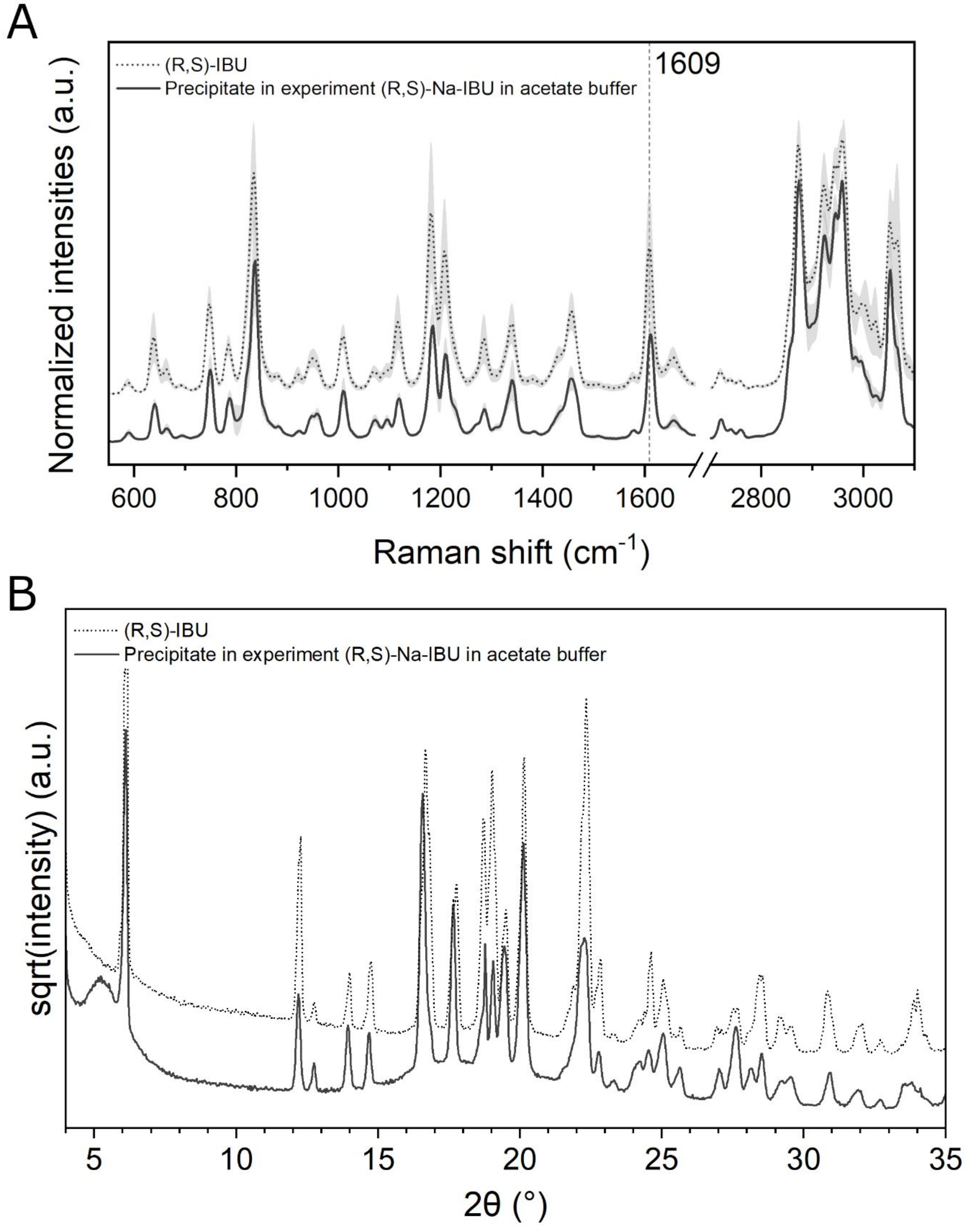

Figure S6. (A) Spontaneous Raman spectra (mean ± SD, n=5) and (B) XRD of collected precipitate in crystallization experiments of (R,S)-Na-IBU (black solid line) in acetate buffer, corresponding to *Scenario 4*. Spontaneous Raman spectrum and reference diffractogram of (R,S)-IBU are presented as dotted lines for reference. The dashed vertical line in subfigure A indicates the Raman peak at 1609 cm^{-1}, characteristic for the crystalline form of (R,S)-IBU. Broad XRPD features found in the collected precipitate measurement are presumably due to the presence of super-saturated solution of amorphous (R,S)-IBU around free acid crystals.

Additional characterization of S-IBU form II

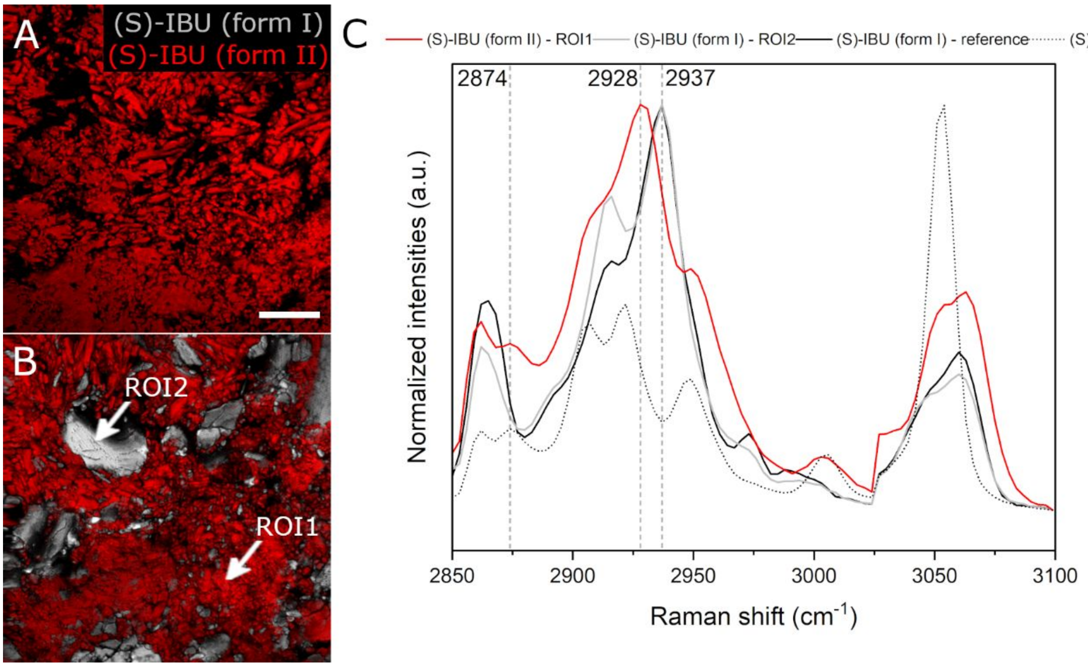

Figure S7. (A-B) False-colored SRS images based on classical least squares (CLS) analysis of image spectra (light grey: (S)-IBU form I, red: (S)-IBU form II), corresponding to the halo in HCl medium (A) and precipitate in acetate buffer (B). Both samples were collected in replicate experiments of *Scenarios 1* and *2*. (C) Spectra of representative regions corresponding to (S)-IBU form II (ROI1) and (S)-IBU form I (ROI2), extracted from SRS image shown in subfigure B. Reference spectra from (S)-IBU form I and (S)-Na-IBU are included for comparison. The CLS library spectra comprised the two (S)-IBU forms and (S)-Na-IBU, but CLS did not classify any pixel as belonging to the salt.

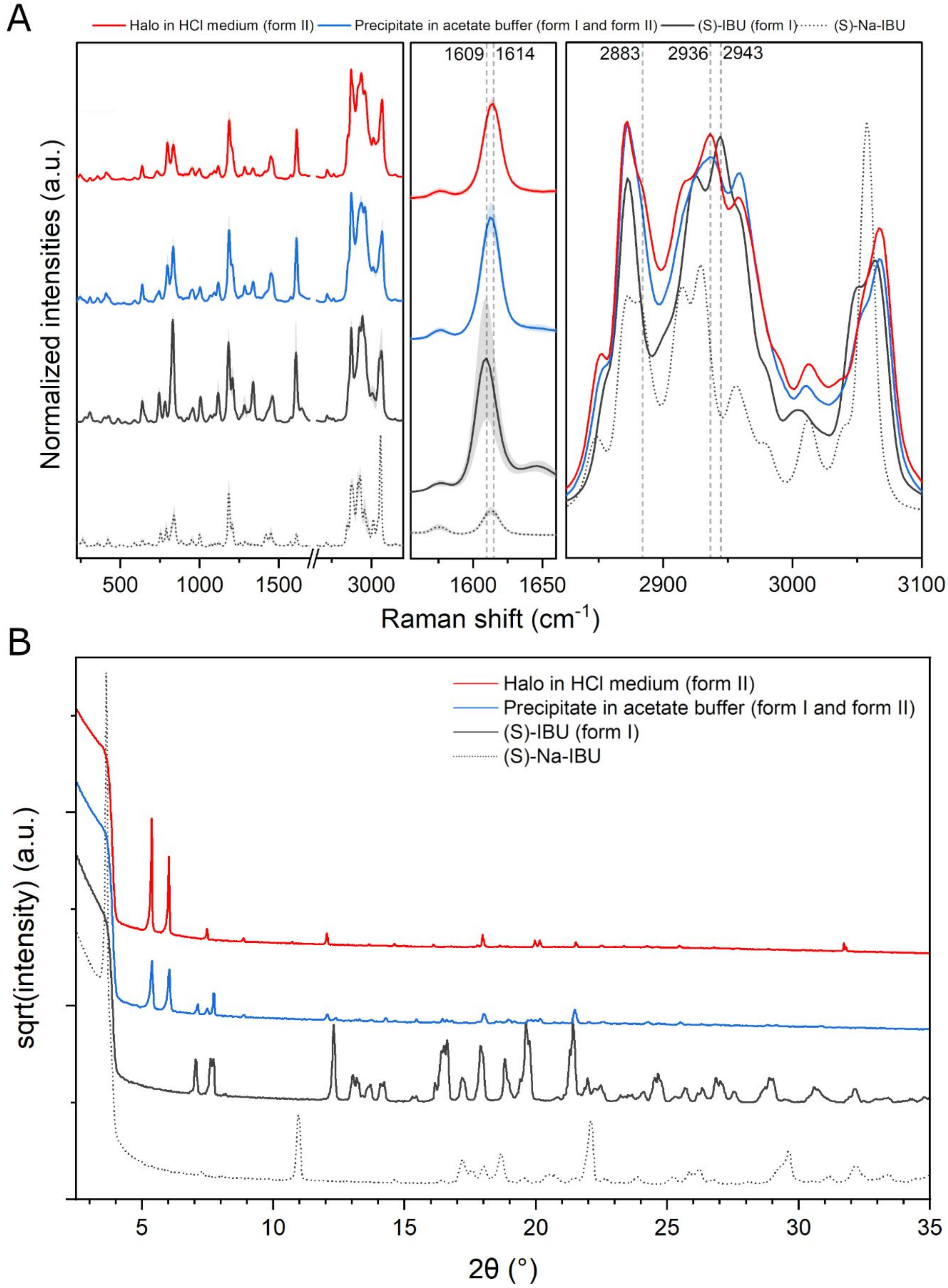

Figure S8. (A) Spontaneous Raman spectra (mean ± SD, n=5) and (B) XRPD diffractograms of collected samples corresponding to the halo in HCl medium and precipitate in acetate buffer. Both samples were collected in replicate experiments of *Scenarios 1* and *2*. Spontaneous Raman spectra and reference diffractograms of (S)-IBU form I and (S)-Na-IBU are also presented for comparison. Dashed vertical lines in subfigure A indicate characteristic spectral features for comparison between form II and form I. The precipitate in acetate buffer appears to be a mixture of S-IBU forms I and II. The halo in HCl medium is composed of the pure S-IBU form II phase, and based on the analysis using Highscore

Plus 5.2, the peak positions do not correspond to any known structures in Crystallography Open Database (COD) or Cambridge Structural Database (CSD).

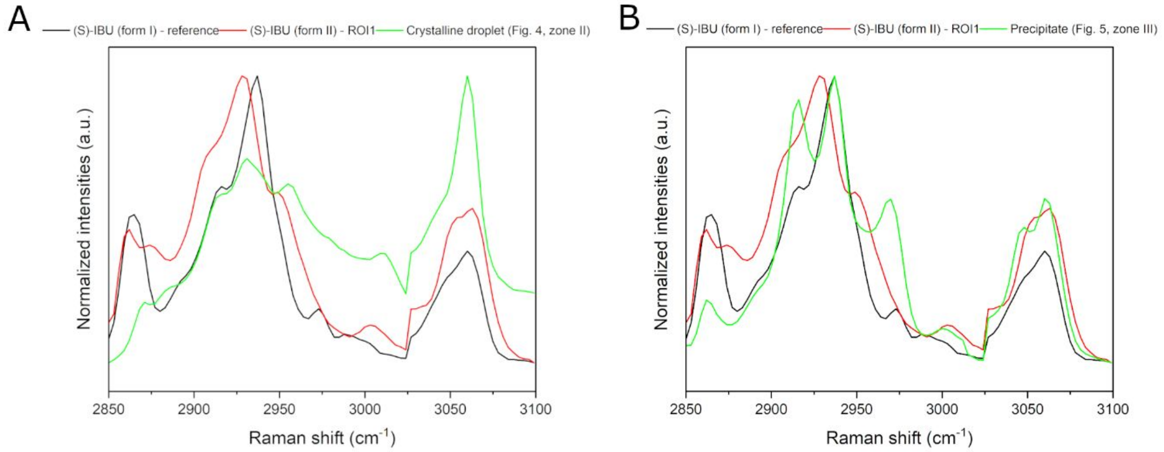

Figure S9. (A) SRS spectrum of a crystalline droplet from the halo in HCl medium (*Scenario 1*, see Fig. 4), as well as spectra of reference (S)-IBU forms I and II. The droplet spectrum does not match any of the known (S)-IBU forms, which suggests a different polymorph. (B) SRS spectrum of precipitate in acetate buffer (*Scenario 2*, see Fig. 5), as well as the reference (S)-IBU forms I and II. The precipitate resembles form I of (S)-IBU, based on peak position analysis. In subfigure B, relative peak intensities vary between (S)-IBU form I and precipitate due to crystal orientation, but peak positions are the same, which indicates that it is the same compound.
